# Management of Schwannoma in the hepatoduodenal ligament: A case report and review of the literature

**DOI:** 10.1097/MD.0000000000018797

**Published:** 2020-01-17

**Authors:** Yan-an He, Chao Yan, Yao Chen, Li-gang Zhu, Ming Cai, Wen-tao Wang

**Affiliations:** a Department of Hepatobiliary Surgery, People's Hospital of Jiangyou, Mianyang; b Department of Liver Surgery & Liver Transplantation Center, West China Hospital of Sichuan University, Chengdu, P.R. China.

**Keywords:** Hepatoduodenal ligament, Multidisciplinary team, S100, Schwannoma

## Abstract

**Rationale:**

Schwannomas are neoplasms that originate from Schwann cells of the peripheral nerve sheath with a low malignant potential. Considering that Schwannomas often occur in the upper extremities, trunk, head, and neck, but in the hepatoduodenal ligament has seldom been reported.

**Patient concerns:**

A 70-year-old man was referred to our hospital for further evaluation of distension in upper abdomen. Abdominal ultrasonography reported that an anechoic mass was found between the pancreatic head and portal vein, which was measured to be about 5.5 × 4 × 4 cm. No blood flow signal was found within the mass by color doppler ultrasound. Subsequently, abdominal contrast enhanced computed tomography revealed that a well-defined round soft-tissue was above the pancreatic head and adjacent to the common heapatic artery, and it had no obvious enhancement in the arterial phase and portal phase.

**Diagnoses:**

Schwannomas in the hepatoduodenal ligament.

**Interventions:**

After the work-up of a multidisciplinary team, a right complete excision was carried out and schwannoma was diagnosed by pathology.

**Outcomes:**

The patient's postoperative course was uneventful, and he left the hospital 10 days after the operation. Additionally, at the time of writing, recurrence was not observed with a follow-up of 17 months.

**Lessons:**

schwannomas in the hepatoduodenal ligament are extremely rare with benign behavior. Surgical resection is the gateway to cure it; however, accurate preoperative diagnosis of the schwannomas in the hepatoduodenal ligament is a huge challenge because neither the clinical symptoms nor the imaging manifestations are specific.

## 1 Introduction

Schwannoma is 1 kind of mesenchymal neoplasms with low malignant potential that composed entirely of Schwann cells.^[1]^ The most common locations of schwannomas are in the upper extremities, trunk, head, and neck, retroperitoneum, mediastinum, pelvis, and peritoneum, but rare in the hepatoduodenal ligaments.^[2]^ According to all the literature worldwide, only 5 patients have been diagnosed as benign schwannoma in hepatoduodenal ligament until now.^[2–6]^ As schwannomas in the hepatoduodenal ligament are normally asymptomatic and often discovered incidentally, preoperative diagnosis is a huge challenge. We tried to present another case and analyzed the clinical and pathological characteristics of schwannoma in the hepatoduodenal ligament through a systematic review of literature.

## 2 Case report

On August 1, 2017, a 70-year-old Chinese male patient was transferred from the department of gastroenterology to the department of hepatobiliary surgery in the People's Hospital of Jiangyou (Jiangyou, Sichuan, China), presenting a complaint of distension in upper abdomen for 1 year. He had a history of inguinal hernia repair. No specific findings were noted in his family history and personal history. Besides, he took no medications.

Physical examination revealed a soft, lax, and non-distended abdomen without evidence of a palpable mass, and no enlarging lymph nodes were identified in the examinable sites. For further diagnosis, laboratory results were normal.

**Figure 1:**
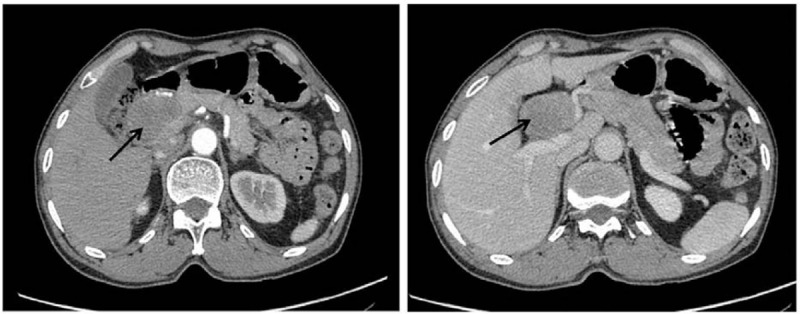
CT scans showing a 5 × 3.5 × 3 cm uniform density mass in the space between the porta hepatis and the stomach (arrow). CT = computed tomography.

Moreover, abdominal ultrasonography (US) (Esaote; Esaote Mylab50; Esaote China Limited, Beijing, China) reported that an anechoic mass was found between the pancreatic head and portal vein, which was measured to be about 5.5 × 4 × 4 cm. No blood flow signal was found within the mass by color doppler ultrasound (Esaote Mylab50). Subsequently, an abdominal computed tomography (CT) scan (GE Lightspeed VCT; Hangwei Tongyong Electric Medicine System Co., Ltd., Beijing, China) was utilized to examine the region. It showed a well-defined round soft-tissue was above the pancreatic head and adjacent to the common hepatic artery, and it had no obvious enhancement in the arterial phase and portal phase (Fig. 1). Based on the findings of the CT scan, the abdominal mass was primarily considered to be a benign tumor, and may be removed.

After the work-up of a multidisciplinary team (MDT) including a gastroenterologist, an oncologist and a radiologist, we came to a decision to treat the patient with exploratory laparotomy. A formal consent was obtained for surgical management.

**Figure 2:**
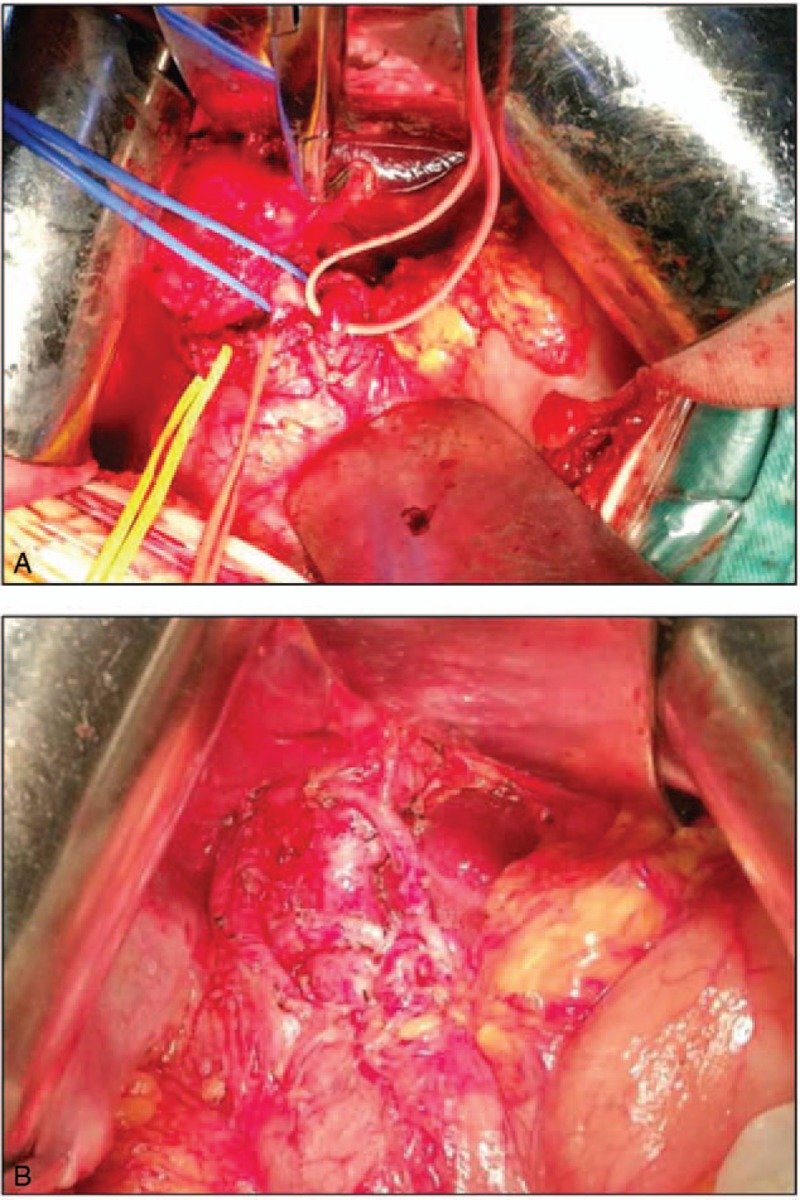
Intraoperative and macroscopic findings. (A) the tumor was found to be located in the hepatoduodenal ligament, (B) the tumor was located above the common bile duct, portal vein and hepatic artery.

We found that the mass surrounded by a fibrous capsule in the hepatoduodenal ligament was located above the common bile duct, portal vein, and hepatic artery (Fig. 2A, B). No invasion of the surrounding tissue was observed and the biliary ducts were not dilated. The tumor blood supply was mainly from the proper hepatic artery and its right and left hepatic artery bifurcation. We separated these tissues around the mass and ligated the tumor blood vessels carefully, then completely resected the mass. Intraoperative frozen-section pathology could not offer an accurate diagnosis but only refered to a spindle cell tumor.

**Figure 3:**
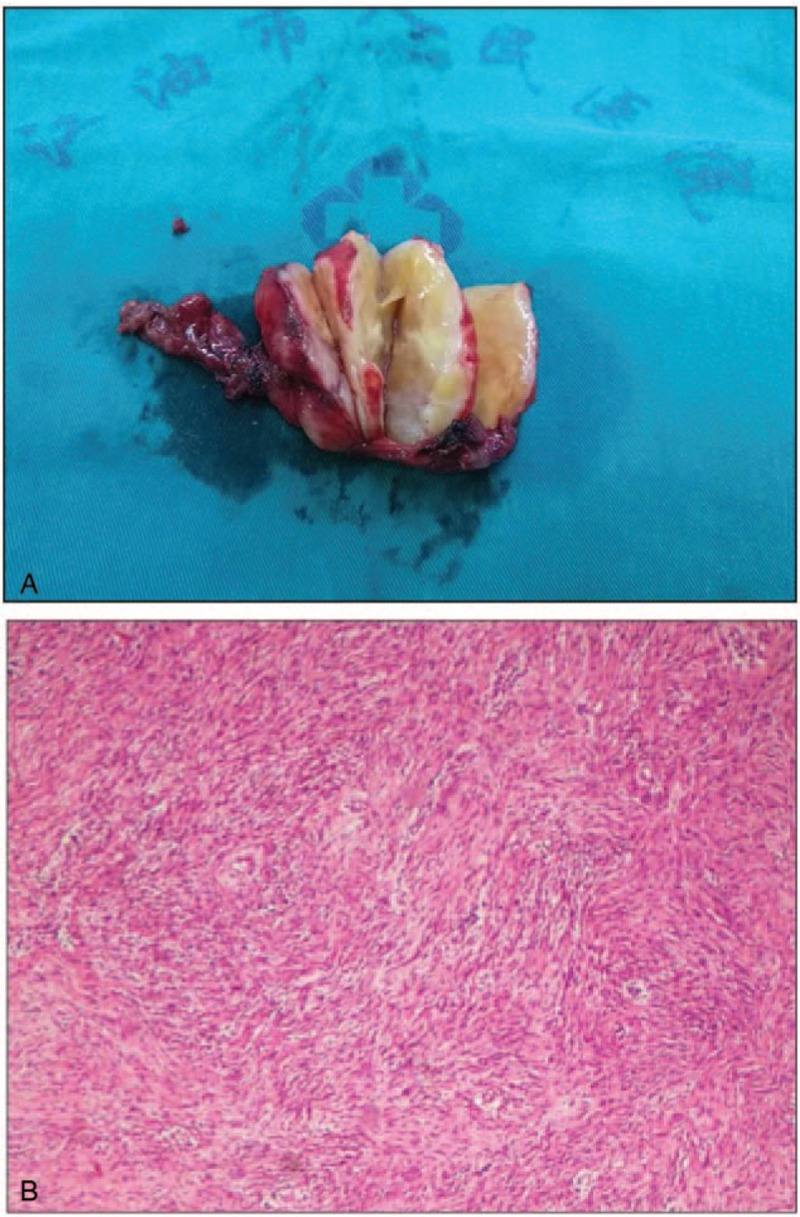
Macroscopic examination. A: The tumor was well encapsulated and the cut surface was yellowish-white in color, B: Microscopic examination showed that the tumor was mainly composed of spindle-shaped cells (HE × 100).

**Figure 4:**
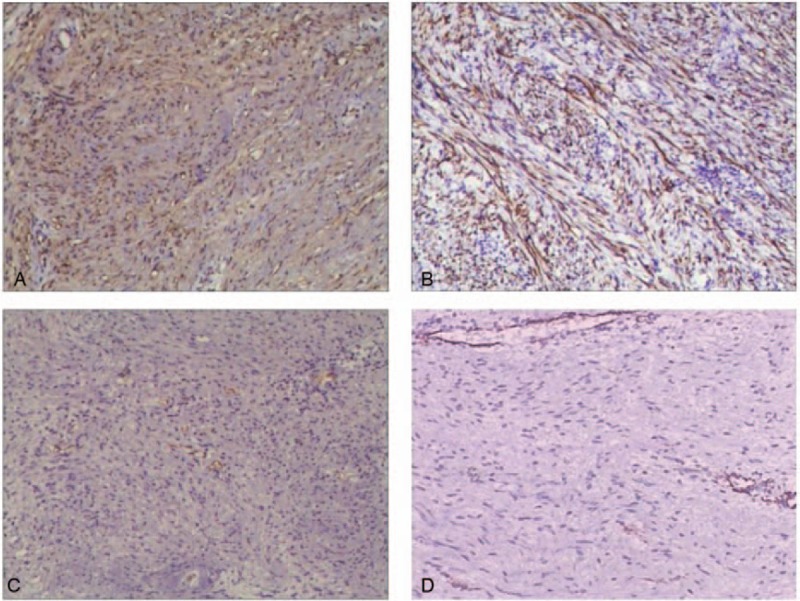
Immunohistochemical showed that protein S-100(A), GFAP(B) were positive, while CD3(C) and smooth muscle actin(SMA) (D) were negative(HE × 100). GFAP = glial fibrillary acidic protein.

The resected specimen was measured to be 5 × 3.5 × 3 cm. Macroscopically, the tumor was well encapsulated and the cut surface was yellowish-white in color (Fig. 3A). Microscopically, the tumor was predominantly composed of spindle-shaped cells without atypia, and compatible with a benign schwannoma which was visible in both hypercellular and hypocellular areas (Fig. 3B). Immunohistochemical investigation showed that protein S100 (Fig. 4A) and glial fibrillary acidic protein (Fig. 4B) were positive, while CD34 (Fig. 4C), CD117 and smooth muscle actin (SMA) (Fig. 4D) were negative. The final diagnosis indicated that was benign schwannoma in the hepatoduodenal ligament.

The patient's postoperative course was uneventful, his liver function blood studies normalized and he left the hospital 10 days after the operation. Furthermore, no recurrence was observed in the follow-ups of following 17 months.

## 3 Literature review

To date, only 5 patients have been diagnosed as schwannoma in the hepatoduodenal ligament from the research of these literatures: Pubmed, EMBASE, and google scholar. The medium age of these patients were 46.5 years old (range: 29–69 years old). Therefore, the patient in our case was the oldest 1 (70 years old). And, all patients were reported with satisfactory results without recurrence. The clinical characteristics of all the 5 cases and our current case are summmarized in Table 1.

**Table 1:**
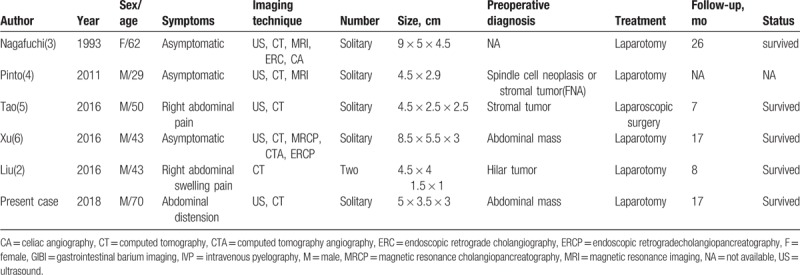
Clinical characteristics of 6 patients with benign schwannoms in the hepatoduodenal ligament.

So far, including our case, there are only 6 cases with schwannomas in the hepatoduodenal ligament are presented, so the understanding on this disease is still limited. The mean age of the patients was 49.5 years old (range 29–70 years old), and the ratio of male and female is 5:1. Patients may exhibit symptoms by compressing adjacent structures such as the bile duct and the gastrointestinal tract. In all cases, 3 patients are asymptomatic and 3 patients were symptomatic. The commonest symptom was abdominal pain (2/6 patients of 33.33%), followed by abdominal distension (1/6 patients or 16.67%). These patients had no symptoms of jaundice, nausea, vomiting, weight loss, and so on.

## 4 Discussion and Conclusion

Schwannomas, also called neurilemomas, neuromas or neurinomas, are neoplasms that originate from schwann cells of the peripheral nerve sheath.^[7]^ Although upper extremities, trunk, head, neck, retroperitomeun, mediastinum, pelvis, and peritoneum are the commonest sites, they may also appear in other areas.^[8]^ The sympathetic and parasympathetic fibers are distributed along the hepatoduodenal ligament, with their branches interwoven into a network, which are the anatomical location of occurrence of the schwannomas in the hepatoduodenal ligament.^[9]^ However, to the best of our knowledge, schwannomas in the hepatoduodenal ligament are extremely rare.

Patients with schwannomas in the hepatoduodenal ligament usually present hepatoduodenal ligament mass on imaging studies. The ultrasound imaging often shows isoechoic or hypoechoic solid masses with well-defined limits.^[5]^ Generally, a CT scan shows a well-defined hypodense heterogenous mass with peripheral enhancement.^[10]^ On magnetic resonance imaging (MRI), the schwannomas typically appear hypointense on T1-weighted images, and appear inhomogeneous and hyperintense on T2-weighted images.^[10]^

Hence, accurate preoperative diagnosis of the schwannomas in the hepatoduodenal ligament is a huge challenge because neither the clinical symptoms nor the imaging manifestations are specific. However, prior to treatment, comprehensive imaging modalities, including US, CT, and MRI, are still essential for establishing a probable diagnosis and determining the lesion limits. In order to provide a definitive diagnosis, fine needle biopsy (FNA) is a good choice.^[9]^ In the 6 cases, the FNA was operated only for 1 patient. However, the results of FNA may be inconsistent with the final pathological results, so the definite diagnosis can only be determined by the histopathology and immunohistochemical examination of the surgical specimens.^[9]^So, after the work-up of MDT, we came to a decision to treat the patient with exploratory laparotomy rather than FNA.

In the 6 cases, most of the tumors were isolated and single, and only 1 patient was with 2 masses.^[2]^ The tumors were varying from 1.5 cm (the largest tumor diameter line) to 9 cm in size, with a median of approximately 5.4 cm. Microscopically, schwannomas are encapsulated tumors and the pattern is alternating Antoni A and B areas with varying relative amounts.^[11]^ The former is hypercellular and characterized by closely packed spindle cells with occasional nuclear palisading and verocay bodies.^[11]^ The latter is hypocellular and is occupied by lossly arranged tumor cells.^[11]^ These 2 kinds of structures often coexist in the same tumor, but mostly with 1 main type. Immunohistochemical staining is strongly positive for S100 and negative for desmin, smooth muscle myosin, SMA, classification determinant (CD)34 and CD117.^[11]^

One documented case of the malignant degeneration in a benign schwannoma has been reported.^[7]^ Therefore, the main treatment of schwannomas is complete mass excision without lymph node dissection.^[9]^ Surgery can not only remove the lesion, but also determine the location and nature of the lesion. In our present case, by laparotomy, we found that the mass was located in the hepatoduodenal ligament and adjacent to important tissues and organs including the common bile duct, proper hepatic artery and portal vein. The tumor vascular supply was mainly from the proper hepatic artery. We successfully separated these tissues around the mass without collateral injury. The overall prognosis of schwannomas in the hepatoduodenal ligament is good after complete excision which requires for noadditional treatments, and in most cases the patients do not relapse.

In conclusion, schwannomas in the hepatoduodenal ligament is a very rare condition with benign behavior. However, to date, its etiology, epidemiology, diagnosis, and treatment are still poor understood. In clinical work, MDT discussion is also a good choice to determine the diagnosis and treatment of patients with hepatic duodenal ligament mass that cannot be diagnosed preoperatively.

## Acknowledgments

We acknowledged the work of management involved in the comprehensive treatments in these individuals (Yu Cai, Yonghong He, Qing Chen, Xing Wang, Bo Tang.).

## Author contributions

**Conceptualization:** Yan-an He, Wen-tao Wang.

**Data curation:** Yan-an He, Li-gang Zhu.

**Formal analysis:** Yan-an He, Yao Chen, Li-gang Zhu.

**Funding acquisition:** Wen-tao Wang.

**Investigation:** Yan-an He, Yao Chen.

**Methodology:** Ming Cai, Wen-tao Wang.

**Project administration:** Ming Cai, Wen-tao Wang.

**Resources:** Wen-tao Wang.

**Software:** Yan-an He, Chao Yan.

**Supervision:** Ming Cai.

**Validation:** Wen-tao Wang.

**Visualization:** Yan-an He, Chao Yan.

**Writing – original draft:** Yan-an He, Chao Yan.

**Writing – review and editing:** Wen-tao Wang.

Yan-an He orcid: 0000-0001-7341-7809.

## References

[1] Yu RS Sun JZ Pancreatic schwannoma: CT findings [J]. Abdom Imaging 2006;31:103–5. 16132429 10.1007/s00261-005-0345-1

[2] Liu J Wang QQ Xie Q Benign schwannoma of the hepatoduodenal ligament: a case report [J]. Int J Clin Exp Med 2016;9:20349–51.

[3] Nagapuchi Y Mitsuo H Takeda S Benign schwannoma in the hepatoduodenal ligament: Report of a case [J]. Surgery Today 1993;23:68–72. 7681709 10.1007/BF00309003

[4] Pinto J Afonso M Veloso R Benign schwannoma of the hepatoduodenal ligament [J]. Endoscopy 2011;43:E195–6. 21590600 10.1055/s-0030-1256354

[5] Tao L Xu S Ren Z Laparoscopic resection of benign schwannoma in the hepatoduodenal ligament: a case report and review of the literature [J]. Oncol Lett 2016;11:3349–53. 27123115 10.3892/ol.2016.4410 PMC4841058

[6] Xu SY Sun K Xie HY Schwannoma in the hepatoduodenal ligament: a case report and literature review. World J Gastroenterol 2016;22:10260–6. 28028376 10.3748/wjg.v22.i46.10260 PMC5155187

[7] Gupta TKD Brasfield RD Strong EW Benign solitary schwannomas (neurilemomas). Cancer 1969;24:355–66. 5796779 10.1002/1097-0142(196908)24:2<355::aid-cncr2820240218>3.0.co;2-2

[8] Abell MR Hart WR Olson JR Tumors of the peripheral nervous system. Hum Pathol 1970;1:503–51. 4330996

[9] Yin SY Zhai ZL Ren KW Porta hepatic schwannoma: case report and a 30-year review of the literature yielding 15 cases. World J Surg Oncol 2016;14:103. 27038921 10.1186/s12957-016-0858-9 PMC4818894

[10] Rha SE Byun JY Jung SE Neurogenic tumors in the abdomen: tumor types and imaging characteristics. Radiographics 2003;23:29–43. 12533638 10.1148/rg.231025050

[11] Xu SY Guo H Shen Y Multiple schwannomas synchronously occurring in the porta hepatis, liver, and gallbladder. Medicine 2016;95:e4378. 27537565 10.1097/MD.0000000000004378 PMC5370792

